# 

**Published:** 1892-03

**Authors:** 


### CONTENTS:

Article I.—Antiseptics and Disinfectants used in Dental Surgery. By Saville H. Hayward, - - - - - 481

" II.—Dislocations—Fractures of the Jaw. By Mr. H. S. Prideaux. - - - - - - - - 494

" III.—One Step in Advance. By Oliver Martin, L. D. S. - 503

" IV.—Dentine. By Mr. Mummery. - - - - - 506

" V.—A Word to Students on the Importance of Mechanical Dentistry. By Guy Harper, L. D. S. - - 512

### EDITORIAL.

The Maryland Dental Law, - - - - - - 514

The Maryland State Dental Association, - - - - 522

### MONTHLY SUMMARY.

Electricity in Tooth Extraction, - - - - - - 523

Two Collateral Cases, - - - - - - - 525

Eucalypto Resorcin, - - - - - - - 526

A Student of Pharmacy, - - - - - - - 526

The Implantation of Artificial Teeth, - - - - - 527

Death from Cocaine Injection, - - - - - - 527

Plaster of Paris Formula, - - - - - - - 528

